# Epidemiology and molecular analyses of respiratory syncytial virus in the 2021–2022 season in northern Italy

**DOI:** 10.3389/fmicb.2023.1327239

**Published:** 2024-01-04

**Authors:** Alessia Lai, Annalisa Bergna, Valentina Fabiano, Carla della Ventura, Giulia Fumagalli, Alessandra Mari, Martina Loiodice, Gian Vincenzo Zuccotti, Gianguglielmo Zehender

**Affiliations:** ^1^Department of Biomedical and Clinical Sciences, University of Milan, Milan, Italy; ^2^Pediatric Department, "Vittore Buzzi" Children's Hospital, Milan, Italy

**Keywords:** respiratory syncytial virus, epidemiology, pediatric cohort, RSV subgroups, RSV genotypes, phylogeny

## Abstract

**Background:**

Human respiratory syncytial virus (RSV) is the leading cause of acute lower respiratory tract infection among infants and young children worldwide, with seasonal peaks in January and February. This study aimed to characterize the RSV samples from a pediatric cohort in the 2021–2022 season in Italy.

**Methods:**

In total, 104 samples were collected from pediatric patients attending the “Vittore Buzzi” Children’s Hospital in Milan, Italy in the 2021–2022 season. RT-PCR and next-generation sequencing were used to discriminate subgroups and obtain whole genomes. Maximum likelihood and Bayesian phylogenetic methods were used to analyze Italian sequences in the European contest and date Italian clusters.

**Results:**

The median age was 78 days, and 76.9% of subjects required hospitalization, with a higher proportion of patients under 3 months of age. An equal proportion of subgroups A (GA2.3.5) and B (GB5.0.5a) was found, with significant differences in length of hospitalization, days of supplemental oxygen treatment, and intravenous hydration duration. Phylogeny highlighted 26 and 37 clusters containing quite the total of Italian sequences for RSV-A and -B, respectively. Clusters presented a tMRCA between December 2011–February 2017 and May 2014–December 2016 for A and B subgroups, respectively. Compared to European sequences, specific mutations were observed in Italian strains.

**Conclusion:**

These data confirmed a more severe clinical course of RSV-A, particularly in young children. This study permitted the characterization of recent Italian RSV whole genomes, highlighting the peculiar pattern of mutations that needs to be investigated further and monitored.

## 1 Introduction

Respiratory syncytial virus (RSV) is the most common cause of acute respiratory infections in infants and children under 2 years of age. It can cause asymptomatic or symptomatic infections ranging from mild to severe, including upper respiratory tract infections (URTI) and lower respiratory tract infections (LRTI; Kaler et al., 2023). A high burden, approximately 60%, of infection is reported in infants younger than 1 year (Piedimonte, 2015). Globally, there are 64 million infections and 160,000 deaths annually from RSV, making this virus the second leading cause of death globally after malaria.^1^^,^^2^

Respiratory syncytial virus shows a seasonal distribution in the temperate regions of the northern hemisphere, including Italy, and most infections generally occur in the period from October/November to March/April, with the peak incidence in January/February, partly overlapping with the epidemiological peak of the flu season (Obando-Pacheco et al., 2018). RSV is classified into A and B subgroups, with wide genetic diversity, especially in the *G* gene, resulting in divergence into many genotypes. Both subgroups circulate simultaneously during the annual epidemic season, although, typically, one predominates each year (Goya et al., 2020; Chen et al., 2022).

Repeated infection is common throughout life (Glezen et al., 1986; Pasittungkul et al., 2022), usually resulting in mild symptoms, but it can also cause serious diseases in older (age ≥ 65 years) or immunocompromised adults and in people with chronic cardiopulmonary disease (Busack and Shorr, 2022). Despite decades of effort, there is no efficacious antiviral for the treatment of the disease; the standard of care remains supportive management only. Palivizumab, an RSV-specific humanized monoclonal antibody, is an available immunoprophylactic agent. It requires multiple administrations over the RSV season and is very expensive; therefore, its use is limited to the highest-risk populations, namely infants born prematurely and those with congenital heart disease, chronic pulmonary disorders, or severe combined immunodeficiency (Garegnani et al., 2021). More recently, the Food and Drug Administration (FDA) approved another monoclonal antibody, nirsevimab, to be used for the prevention of RSV lower respiratory tract disease (LRTD) in neonates and infants born during or entering their first RSV season and in children up to 24 months of age who remain vulnerable to severe RSV disease through their second RSV season. The efficacy and safety of nirsevimab have been evaluated in three clinical trials (Griffin et al., 2020, Domachowske et al., 2022, Hammitt et al., 2022). Moreover, even more recently, the FDA also approved a vaccine, Abrysvo, to be administered as a single intramuscular injection to pregnant women at 32–36 weeks gestational age to prevent LRTD and severe LRTD caused by RSV in infants from birth through 6 months of age (Fleming-Dutra et al., 2023).

The lack of an epidemiological surveillance system of RSV in Italy has led to non-constant data flows, suggesting the prevalence of RSV infections between 5 and 13% among influenza-like illnesses (ILIs), varying by the winter season and location latitude (Pellegrinelli et al., 2020; Tramuto et al., 2021). Since the pandemic emergency due to SARS-CoV-2, information relating to RSV has been collected by the national system for the epidemiological and virological surveillance of influenza, InfluNet.^3^ Due to the implementation of restrictive measures for the prevention and control of the SARS-CoV-2 pandemic, in particular, physical distancing, the use of masks, and the suspension of educational activities, the 2020–2021 season was characterized by a reduced circulation of RSV and other respiratory pathogens (Chuang et al., 2023).

In fact, during the following season, 2021–2022, cases started to increase, with a peak incidence reported 2 months earlier than expected and numerous outbreaks recorded in many countries, including Italy (Weinberger Opek et al., 2021; Williams et al., 2021).

The genotypic information about the virus circulating in Italy is scarce and frequently limited to single genomic portions. Since no data are currently available on the genomic epidemiology of this recent epidemic in our country, the aim of this project is the molecular characterization of the RSV samples in a pediatric cohort, collected at the Vittore Buzzi Children’s Hospital of Milan, and their correlation with clinical and demographic data. In the present study, the phylogenetic relationship and the sequence diversity of the full-length genomes from RSV samples collected in Italy were compared with those from other European countries.

## 2 Methods

### 2.1 Patients

A consecutive series of 104 RSV-positive patients whose nasopharyngeal swab specimens were available, attending Vittore Buzzi Children’s Hospital in Milan between November 2021 and January 2022, were enrolled. No additional samples were required, and those analyzed were obtained using leftover samples of diagnostic testing. For all participants, informed consent was obtained from the parents or legal guardians. All data used in this study were previously anonymized as required by the Italian Data Protection Code (Legislative Decree 196/2003) and the general authorizations issued by the Data Protection Authority. The study was conducted in compliance with Good Clinical Practice^4^ and the Declaration of Helsinki.

### 2.2 Study design

For each patient, the antigenic subgroup (RSV-A or RSV-B) of the infecting virus was determined by RT-PCR (described below).

The whole viral genomes were then obtained by next-generation sequencing (NGS) using specific protocols for each viral subgroup (described in detail below), and the infecting genotype was then recognized by phylogenetic analysis.

Two datasets were then assembled, one for each viral subgroup (RSV-A and -B datasets), including the patients’ genomes with a set of whole-genome sequences of the same genotypes available in public databases at the moment when the study was performed and for which the sampling date and location were known. To limit the size of the datasets, only genomes obtained in Europe were included in the analyses.

The two datasets were then analyzed to find statistically supported clusters containing more than two sequences; subsequently, clusters were analyzed and dated by maximum likelihood in order to reconstruct the origin of the clusters, including Italian isolates.

Finally, the phylodynamics of RSV were studied by means of a Bayesian approach, limiting the analysis only to clusters, including Italian sequences.

#### 2.2.1 RSV-A and RSV-B subgroup assignments

##### 2.2.1.1 Real-time RT-PCR assay for viral subgroup assignment

Viral RNA from nasopharyngeal swab samples was manually extracted with the QIAamp Viral RNA Mini Kit (QIAGEN, Hilden, Germany) following the manufacturer’s instructions. RNA was eluted in 50 μL of water. Specimens and RNA were stored at −80°C until use. A modified version of the duplex rRT-PCR assay proposed by Wang et al. (2019) was developed to distinguish between the RSV subgroups A and B targeting conserved regions in the RSV nucleoprotein (*N*) gene. To minimize overlap in dye emission spectra, the RSV-A and -B probes were labeled at the 5′-end with 6-carboxyfluorescein (FAM) and hexachloro-fluorescein (HEX), respectively. Probes were internally quenched with Black Hole Quencher 1. Primer/probes were synthesized by Integrated DNA Technologies (IDT, Coralville, United States), and probes were HPLC-purified. Luna^®^ Universal One-Step RT-qPCR (New England BioLabs, Ipswich, MA, United States).

Briefly, the assay was performed in 20 μL reactions containing 0.2 μM forward and reverse primers, 0.10 μM RSV-A probe, 0.10 μM RSV-B probe, and 5 μL of extracted RNA on an *CFX* Connect Real-Time PCR System instrument (Bio-Rad, Hercules, United States) using the Luna^®^ Universal One-Step RT-qPCR kit (New England BioLabs, Ipswich, United States). Thermocycling conditions consisted of 10 min at 55°C for reverse transcription, 1 min at 95°C for the activation of the Taq polymerase, and 45 cycles of 15 s at 95°C and 1 min at 55°C.

A specimen was considered positive if a defined fluorescence curve crossed the auto-threshold setting within 45 cycles.

#### 2.2.2 RSV whole-genome amplification and sequencing

The complete RSV genome of each subgroup was covered with 10 overlapping subgroup-specific amplicons with an average size of 1.5–2 kb using the Qiagen One-Step RT-PCR kit (Qiagen, Hilden, Germany) according to a modified version of the methodology proposed by Wang et al. (2022). The RT-PCR reaction was performed in a 25-μL reaction system containing 2 μL of template RNA, 0.75 μL of each 10 μM primer, 5 μL of 5× RT-PCR buffer, 1 μL of dNTP (10 mM), 1 μL of enzyme mix, and 14.5 μL of nuclease-free water. Thermocycling conditions consisted of 30 min at 50°C for reverse transcription, 95°C for 15 min to inactivate the reverse transcriptase enzyme, followed by 45 cycles of 94°C for 30 s, 54°C for 30 s, and 72°C for 2 min 15 s, with a final extension step at 72°C for 7 min for RSV-A and at 55°C for 40 s for the annealing of RSV-B samples. Amplicons were run on 1.3% agarose gels to confirm amplification and purified using a 1.8× volume of Agencourt AMPure XP Beads (Beckman Coulter).

Amplicons were pooled at equal concentrations, and libraries were prepared using Illumina DNA Prep and IDT Illumina DNA/RNA UD Index Kit (Illumina, San Diego, CA, United States). Library concentration was determined with the Invitrogen Quant-iT Picogreen dsDNA assay (Fisher Thermo Scientific, Waltham, MA, United States). The resulting libraries were normalized and pooled for sequencing using a 2 × 200 cycle paired-end sequencing protocol. Reads were mapped to a reference sequence (EPI_ISL412866 and EPI_ISL1653999 for RSV-A and -B, respectively) using the Geneious Prime software v. 11.1^5^ to obtain consensus sequences.

#### 2.2.3 RSV genotype assignment

The genotype assignment was obtained by aligning the *G* sequences obtained from our patients with international sequences with known RSV genotypes available on public databases (*n* = 1,269 RSV-A; *n* = 1,847 RSV-B). Genotype assignment was also confirmed by submitting whole viral genomes to Nextclade^6^ (Aksamentov et al., 2021).

Genotype/subgenotype/lineage definition was based on the nomenclature proposed by Goya et al. (2020) and Ramaekers et al. (2020).

#### 2.2.4 RSV datasets

One dataset for each subgroup (RSV-A and -B datasets) of complete genomes was built, including European sequences, obtained by selecting available strains on GISAID^7^ or GenBank,^8^ carrying the same genotype of Italian strains for the identification of transmission clusters (*n* = 359 RSV-A; *n* = 806 RSV-B). The sequences included in the European datasets were collected between November 2012 and April 2022 and between December 2013 and November 2022 for A and B subgroups, respectively.

The datasets were analyzed by maximum likelihood (see below in the next section for phylogenetic analysis), and the statistically supported clusters, including more than two sequences, were identified by using the Cluster Pickers v.1.2.3 software using 90% bootstrap support and a mean genetic distance of 1% as thresholds. Only clusters including at least one Italian sequence were selected and classified as mixed (M), containing both Italian and non-Italian isolates in different proportions; pure Italian (IT), including only Italian genomes; or singleton (S), containing only a single Italian genome interspersed within non-Italian sequences.

#### 2.2.5 Phylogenetic analysis by maximum likelihood method

Alignment of multiple sequences was obtained using MAFFT,^9^ and the alignment was manually edited by the BioEdit v. 7.2.6.1 program.^10^ Sequences were trimmed at the ends to obtain genomes of the same length (14,931 and 15,197 bp for A and B subgroups, respectively). The maximum likelihood trees of both the genotyping and European datasets were estimated using IQ-TREE v. 1.6.12.^11^ The GTR + F + R6 (general time reversible + empirical base frequencies + six number of categories) model, selected by the program, was used, and 1,000 parametric bootstrap replicates were performed to support the nodes (≥50% bootstrap support).

Trees were visualized and edited in FigTree v. 1.4.4.^12^ Using the bioinformatics pipeline for phylodynamics analysis and the interactive visualization platform Nextstrain, we obtained a dated tree using the annotated file obtain after cluster identification (Hadfield et al., 2018).

A root-to-tip regression analysis was made using TempEst^13^ in order to investigate the temporal signal of the datasets.

#### 2.2.6 Genomic distances and amino acid analysis

The MEGA X program was used to evaluate the genetic distance between and within Italian and European strains and between and within clusters on full-length genomes, with variance estimation performed using 1,000 bootstrap replicates. Amino acid changes were evaluated using the same reference sequences used for mapping using Nextclade. All of the genes were tested for selection pressure using Datamonkey.^14^

#### 2.2.7 Phylodynamic analysis

The largest clusters, including Italian strains, were used for the Bayesian analyses. Bayesian analysis was performed by BEAST v. 1.10.4^15^ with the same substitution model employed for the previously described analyses. The generalized path sampling (PS) and stepping stone (SS) marginal likelihood estimators were used to determine the best fitting clock between strict and relaxed under the Bayesian skyline plot (BSP) model.

MCMC analyses were run for 30 million generations and sampled every 3,000. Convergence was assessed by estimating the effective sampling size (ESS) after applying a 10% burn-in through Tracer v.1.7 software,^16^ accepting ESS of at least 200. The uncertainty of estimates was indicated with 95% highest prior density (HPD) intervals.

The final tree is selected based on the maximum posterior probability (pp) value after performing a 10% burn-in using Tree Annotator v.10.4 software (included in the BEAST package). Posterior probabilities greater than 0.7 were considered significant. Finally, all trees were visualized and edited in FigTree v. 1.4.4 (see text footnote 12).

#### 2.2.8 Statistical analysis

Descriptive analyses of demographic and clinical data are presented as median and interquartile range (IQR) when continuous and as frequency and proportion (%) when categorical. Parametric tests (*t*-test and ANOVA), non-parametric tests (Mann–Whitney and Kruskal–Wallis), and the Pearson χ^2^ test (or Fisher exact test, when necessary) were used to compare normally distributed, non-normally distributed continuous, and categorical variables of patients, respectively. Significance was established at a value of *p* < 0.05. Data analysis was performed using IBM SPSS Statistics version 25.

## 3 Results

### 3.1 Study population and subgroup RSV-A/-B characterization

Table 1 shows the characteristics of enrolled patients in the whole population and after stratification according to the virus subgroup. Most of the subjects belonged to male sex (61/104, 58.7%), with a median age of 78 days (IQR: 45–195). A large proportion of subjects required hospitalization (80/104, 76.9%); in particular, this proportion was significantly higher among those younger than 3 months of age (Table 2). The median duration of hospitalization was 8 days (IQR: 5–10). A total of 73.8% (59/80) of the hospitalized patients required oxygen supplementation for a median duration of 5 days (IQR: 3–7). Clinical complications were observed in 22 subjects (21.1%); all these patients were hospitalized. The most frequently observed complication was acute respiratory failure (10/22, 45.5%). Five hospitalized subjects (6.3%) presented a coinfection with SARS-CoV-2, one with bocavirus and SARS-CoV-2 and one presented a bacteremia caused by *Enterococcus faecalis* (1.3%).

**Table 1:** Characteristics of studied subjects stratified according to subgroup.

Characteristics	Overall	HRSV-A	HRSV-B	*p*
Study population, *n* (%)	104	52 (50)	52 (50)	
Sex, *n* (%)				
Male	61 (58.7)	29 (55.8)	32 (61.5)	*0.550*
Female	43 (41.3)	23 (44.2)	20 (38.5)	
Age median days (IQR)	78 (45–195)	109 (53–263)	66 (31–116)	*0.007*
Age groups, *n* (%)				
≤3 months	58 (55.8)	25 (48.1)	33 (63.5)	*0.016*
3–6 months	19 (18.3)	7 (13.5)	12 (23.1)	
6 months–1 year	17 (16.3)	11 (21.1)	6 (11.5)	
>1 year	10 (9.6)	9 (17.3)	1 (1.9)	
Non-hospitalized	22 (21.1)	12 (23.1)	10 (19.2)	*0.80*
Hospitalized	80 (76.9)	39 (75)	41 (78.8)	
Days of hospitalization median (IQR)	8 (5–10)	8 (7–10)	6 (5–9)	*0.05*
≤7 days	33 (31.7)	23 (36.1)	20 (60.6)	*0.041*
≥8 days	36 (34.6)	23 (63.9)	13 (39.4)	
O_2_ supplementation				
Yes	59 (56.7)	32 (61.5)	27 (13.5)	*0.490*
No	20 (19.2)	19 (36.5)	23 (44.2)	
Days of O_2_ median (IQR)	5 (3–7)	6 (5–8)	4 (2–6)	*0.003*
≤6 days	41 (39.4)	18 (60)	23 (85.1)	*0.034*
≥7 days	16 (15.4)	12 (40)	4 (14.8)	
Undereating				
Yes	44 (42.3)	21 (53.8)	21 (52.5)	*0.904*
No	57 (54.8)	18 (46.1)	19 (47.5)	
Hydration				
Yes	30 (28.8)	16 (42.1)	14 (38.9)	*0.079*
No	44 (42.3)	22 (57.9)	22 (61.1)	
Days of hydration median (IQR)	3 (2–5)	4 (3–6)	2 (2–3)	*0.018*
≤6 days	21 (20.2)	11 (84.6)	10 (100)	*0.179*
≥7 days	3 (2.9)	3 (23.1)	0 (0)	
Complications				
Yes	22 (21.1)	11 (28.2)	11 (27.5)	*0.944*
No	57 (54.8)	28 (71.8)	29 (72.5)	

IQR, Interquartile range.

**Table 2:** Proportion of hospitalized subjects in subjects of different ages.

	Hospitalization		*p* value
	Yes % (*n*)	No % (*n*)	
≤3 months	86.0 (49)	14.1 (8)	0.019
3–6 months	78.9 (15)	21.1 (4)	
6 months–1 year	54.5 (6)	63.6 (7)	
>1 year	76.9 (10)	23.1 (3)	0.037
≤3 months	86.0 (49)	14.1 (8)	
>3 months	68.9 (31)	31.1 (14)	

Fifty-two (50%) of the 104 patients included in the study were assigned to viral subgroup A and 52 (50%) to subgroup B, indicating that viral subgroups were equally distributed in our case file.

When subjects were stratified according to the viral subgroup, a significantly longer duration of hospitalization (8 vs. 6, *p* = 0.05), oxygen therapy (6 vs. 4, *p* = 0.003), and hydration (4 vs. 2, *p* = 0.018) was observed in subgroup A patients compared to subgroup B patients. Moreover, subjects of subgroup A showed a significantly higher median age (109 vs. 66, *p* = 0.007) and a lesser proportion of infants younger than 3 months (48.1 vs. 63.5, *p* = 0.016) compared to subgroup B. No differences were observed in the distribution of gender, the proportion of hospitalization, feeding difficulties, and complications.

### 3.2 RSV whole-genome sequencing and genotype/clade assignment

Whole viral genome was successfully sequenced in 88 of the 104 nasopharyngeal swab samples (84.6%), of which 49 samples were RSV-A and 39 RSV-B, with specific sequencing success rates of 94.2% (49/52) and 75% (39/52), respectively. RSV genotyping analysis showed that all subgroup A strains belonged to genotype GA2, subgenotype GA2.3, and lineage GA2.3.5 on the basis of *G* gene sequences (Goya et al., 2020) and to genome-clade A23 on the basis of the whole genomes (Ramaekers et al., 2020). Similarly, all subgroup B strains belonged to genotype GB5, subgenotype GB5.0 and lineage GB5.0.5a, and genome-clade B6.

### 3.3 Cluster analysis

The phylogenetic analysis of the whole RSV-A dataset showed that 96.9% (348/359) of genomes of lineage GA2.3.5 sampled in Europe, available on public databases, were grouped in a total of 26 significant clusters (bootstrap = 0.83–100), five of which (clusters #5, 12, 14, 25, 26) included Italian isolates.

RSV-B dataset analysis showed that 99.1% (799/806) of lineage GB5.0.5a genomes isolated in Europe are available on public databases and are grouped in 37 significant clusters (bootstrap = 0.51–100). Two Italian sequences were scattered throughout the tree, while all the other 37 Italian genomes were included in six clusters (clusters #2, 13, 15, 23, 24, and 28) with other European sequences (see more detailed description of the sampling locations of the sequences included in clusters below).

### 3.4 Analysis of the RSV genetic/genomic distances

#### 3.4.1 RSV-A dataset (GA2.3.5 lineage)

The whole RSV-A genome showed an overall mean p-distance of 0.0089 sub/site (standard error [*SE*]: 0.0003), corresponding to a mean nucleotide difference per pairwise comparison of 119.27 (*SE*: 4.58).

The synonymous distance (dS) was approximately 1 log greater than the non-synonymous distance (Grubaugh et al., 2019; Table 3A).

**Table 3:** Genetic distances evaluated on whole genomes and the *G* gene among RSV-A and -B strains (part A) and within and between clusters (part B).

A
		*p*-distance (*SE*^a^)	dS (*SE*)	dN (*SE*)	dN/dS
RSV-A	Whole genome	0.0089 (0.0003)	0.032 (0.002)	0.0027 (0.0002)	0.08
	*G* gene	0.022 (0.0021)	0.046 (0.001)	0.015 (0.002)	0.3
RSV-B	Whole genome	0.0054 (0.0002)	0.018 (0.0008)	0.0019 (0.1)	0.10
	*G* gene	0.014 (0.001)	0.024 (0.002)	0.011 (0.002)	0.5

B
		*p*-distance within (IQR^b^)	*p*-distance between (IQR)
RSV-A	Whole genome	0.0035 (0.0022–0.0045)	0.0088 (0.0021–0.0103)
	*G* Gene	0.0088 (0.007–0.0098)	0.0226 (0.0022–0.0036)
RSV-B	Whole genome	0.0026 (0.0016–0.0038)	0.0057 (0.0048–0.0070)
	*G* Gene	0.0066 (0.0037–0.0088)	0.015 (0.015–0.017)

^a^SE: Standard error. ^b^IQR: Interquartile range.

Considering only the *G* sequences, the overall mean *p*-distance was 0.0221, with dS still being higher than nucleotide distance (dN), although at a lower difference than in whole genomes (dN/dS = 0.3; Table 3A).

The independent analysis of a total of nine representative clusters, including >10 genomes, showed a median *p*-distance within and between clusters of 0.0035 sub/site and 0.0088 on the whole genome, and 0.0088 and 0.0226 on the *G* sequences, respectively (Table 3B).

#### 3.4.2 RSV-B dataset (GB5.0.5a lineage)

The analysis of the genomic distance of RSV-B dataset showed a mean *p*-distance of 0.0054 sub/site, corresponding to a mean difference of 66.3 (*SE*: 2.53) nucleotides per pairwise comparison. The synonymous distance was one log higher than the non-synonymous one. Focusing on *G* sequences, the overall *p*-distance was 0.014, with a dN/dS ratio still in favor of dS (dN/dS = 0.5; Table 3A).

The analysis of the 16 largest clusters involving more than 10 sequences showed a median genomic distance within clusters of 0.0021 and between clusters of 0.0057 (Table 3B).

Considering the most representative 16 clusters with ≥ 10 sequences, the median *p*-distance within clusters was 0.0026 and between clusters was 0.057 for whole genomes and 0.0066 and 0.015 sub/site, respectively, considering only *G* sequences (Table 3B).

### 3.5 Amino acid heterogeneity

#### 3.5.1 RSV-A dataset

**Figure 1:**
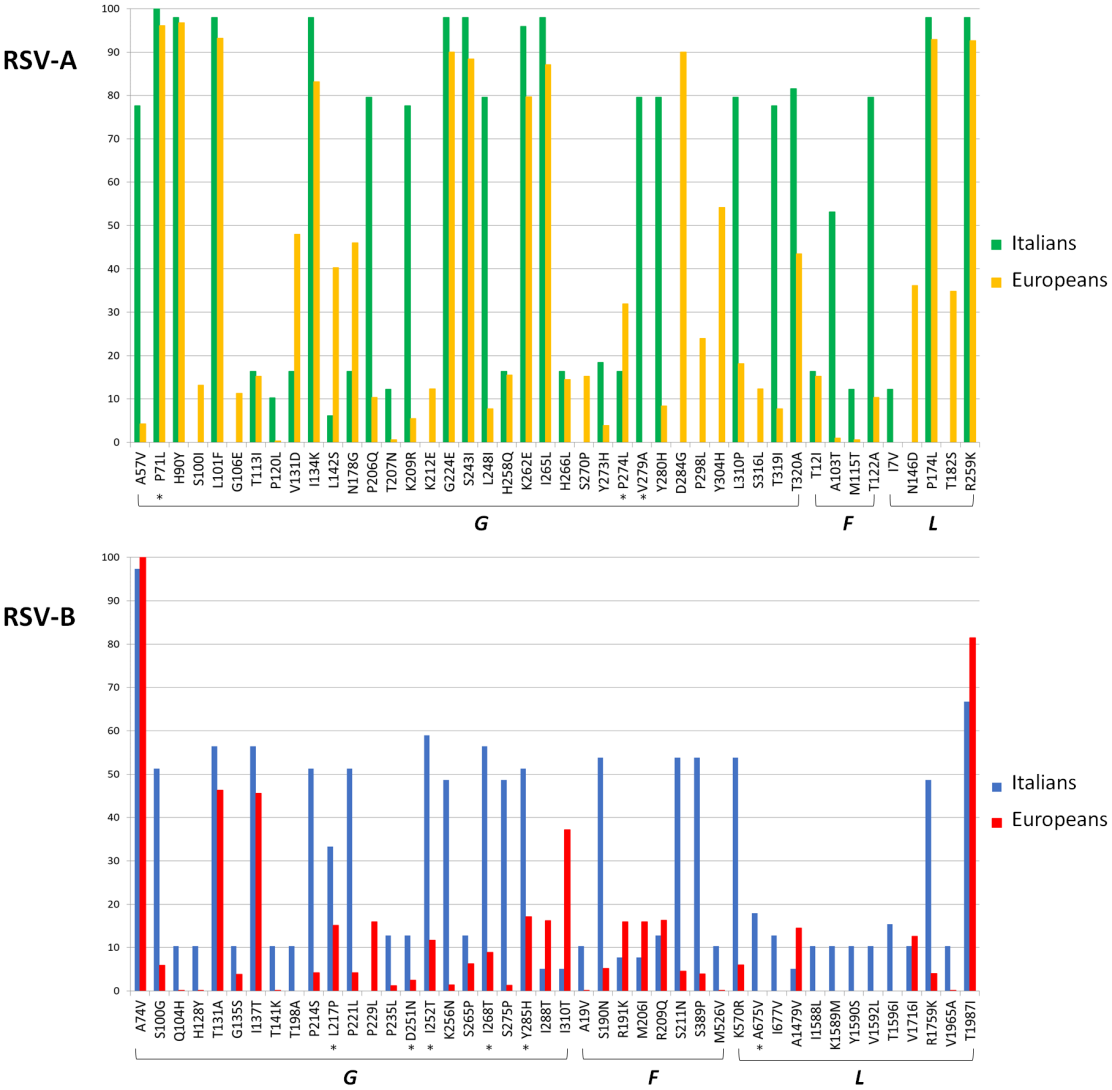
Amino acid substitutions found with a frequency of ≥10% in the G, F, and L genes in RSV-A and -B datasets among Europeans and Italians. Asterisks indicated that the sites were under significant positive selective pressure.

A total of 69 amino acid substitutions were present with a frequency of ≥ 10% in the analyzed Italian or European sequences (Supplementary Table S1) of RSV-A. Figure 1 represents the frequency of the G, F, and L protein substitutions in the Italian and European genomes. Most of these substitutions (*n* = 35, 50.7%) were present in the *G* gene, followed by the *L* gene (*n* = 19, 27.5%). The analysis of the selective pressure showed a total of three sites under significant positive selective pressure (3/69, 4.3%), all located in the G protein (P71L, P274L, and V279A, indicated by an asterisk in Figure 1).

Some substitutions (*n* = 9) were observed only in Italian compared to European genomes, including one of those (V279A) under positive selection (Supplementary Table S1). However, 15 substitutions observed in European strains were not detected in the Italian ones.

#### 3.5.2 RSV-B dataset

A total of 53 amino acid substitutions were present in more than 10% of the Italian and/or other European isolates (Supplementary Table S1; Figure 1) of RSV-B, of which 43.4% (*n* = 23) and 24.5% (*n* = 13) were located in the *G* and *L* genes.

Six out of the 53 sites (11.3%) were under significant positive selection: 5 in the G protein and 1 in the L protein.

Seventeen substitutions were observed only in Italian isolates, while only one mutation present in other European isolates was not detected in Italian sequences. In particular, Italian genomes in cluster #28 presented five specific mutations in the *L* gene.

### 3.6 RSV-A and RSV-B dated phylogenies

For both datasets, root-to-tip regression analysis of the temporal signal revealed an association between genetic distances and sampling days (correlation coefficients of 0.88 and 0.73 and a coefficient of determination [R2] of 0.79 and 0.53 for RSV-A and -B, respectively).

#### 3.6.1 RSV-A dataset

**Figure 2:**
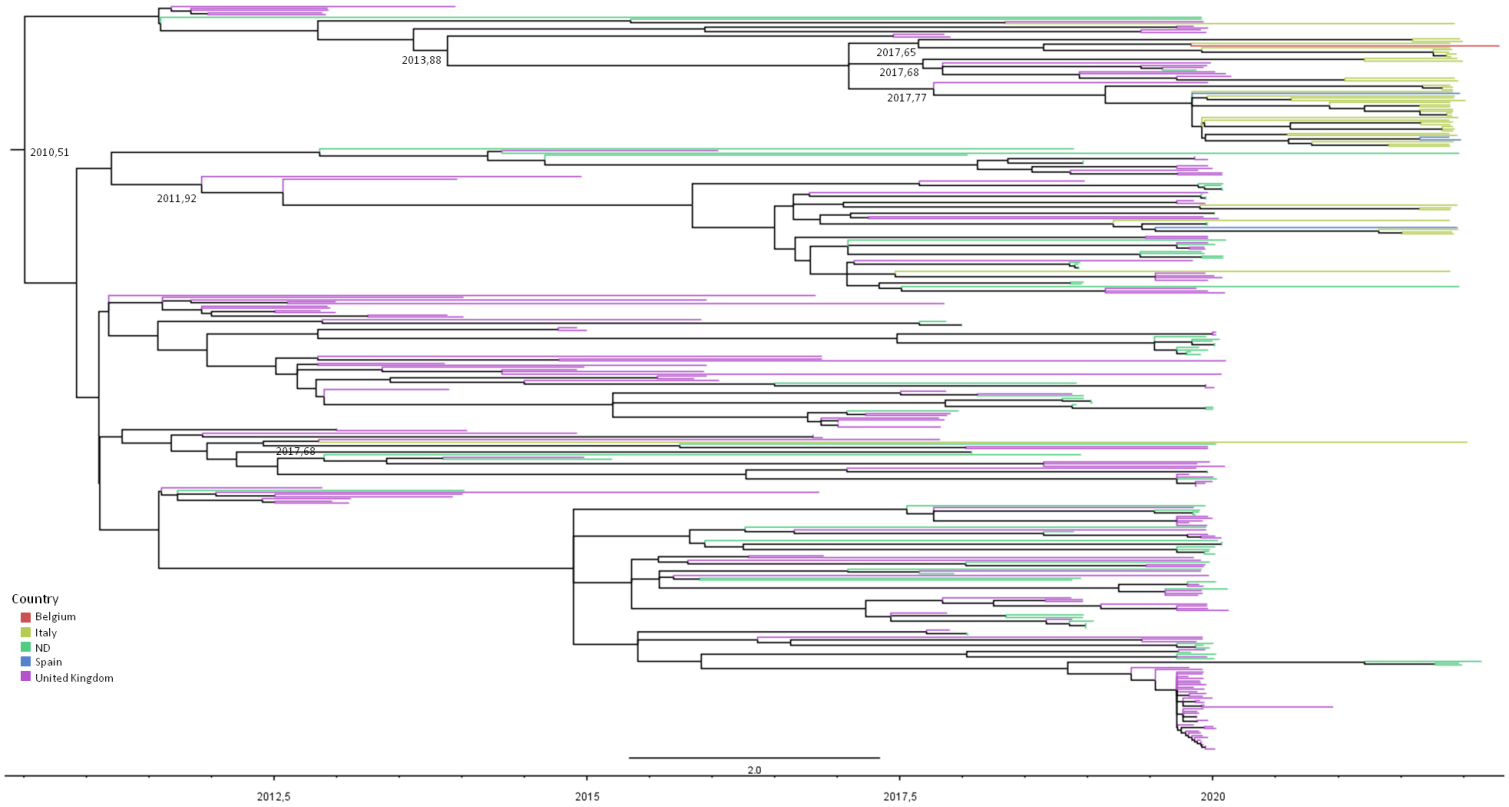
Maximum likelihood dated tree of RSV-A based on the whole genome. The branches’ colors represent the location (described in the legend). The time scale in fraction of year is detailed at the bottom. tMRCA of clusters involving Italians are reported.

Figure 2 represents the dated phylogenetic tree of RSV-A significant clusters obtained by maximum likelihood analysis, showing a tree root dating to mean July 2010 (95% HPD: December 2009–September 2010), which corresponds to the most likely origin of the GA2.3.5 lineage estimated on our dataset.

The majority of the Italian patients’ genomes (38/48, 79.2%) were included in two nested clades (and #26) encompassing also isolates from the United Kingdom, Spain, and Belgium, sharing an MRCA dating between September 2016 and June 2017 (mean February 2017).

Nine (18.7%) isolates were grouped into cluster #5, encompassing also United Kingdom and Spain sequences, whose tMRCA was estimated to be between July 2011 and April 2012 (mean date: December 2011). The two remaining isolates were included in clades #14 and #12 as singletons with other European sequences (Table 4).

**Table 4:** Composition and tMRCA estimation with the relative confidence intervals of clusters of the RSV-A and -B datasets.

Cluster #		Number of sequences	Number of Italians, *n* (%)	tMRCA	95% HPD
RSV-A	5	58	9 (15.5)	02/12/2011	23/07/2011–22/04/2012
	12	3	1 (33.3)	07/05/2015	15/05/2014–27/10/2015
	14	2	1 (50)	10/11/2012	29/08/2012–29/08/2013
	25	41	30 (73.2)	25/08/2017	08/04/2017–11/01/2018
	26	10	8 (80)	05/09/2017	11/05/2017–07/01/2018
RSV-B	2	4	3 (75)	03/08/2012	06/03/2012–06/03/2013
	13	144	1 (0.7)	29/12/2014	15/06/2014–15/04/2015
	15	188	5 (2.6)	29/05/2014	01/01/2014–03/01/2015
	23	4	2 (50)	02/12/2015	05/07/2015–02/06/2016
	24	26	7 (26.9)	26/10/2015	09/06/2015–11/04/2016
	28	50	21 (42)	16/12/2016	21/05/2016–23/06/2017

#### 3.6.2 RSV-B dataset

**Figure 3:**
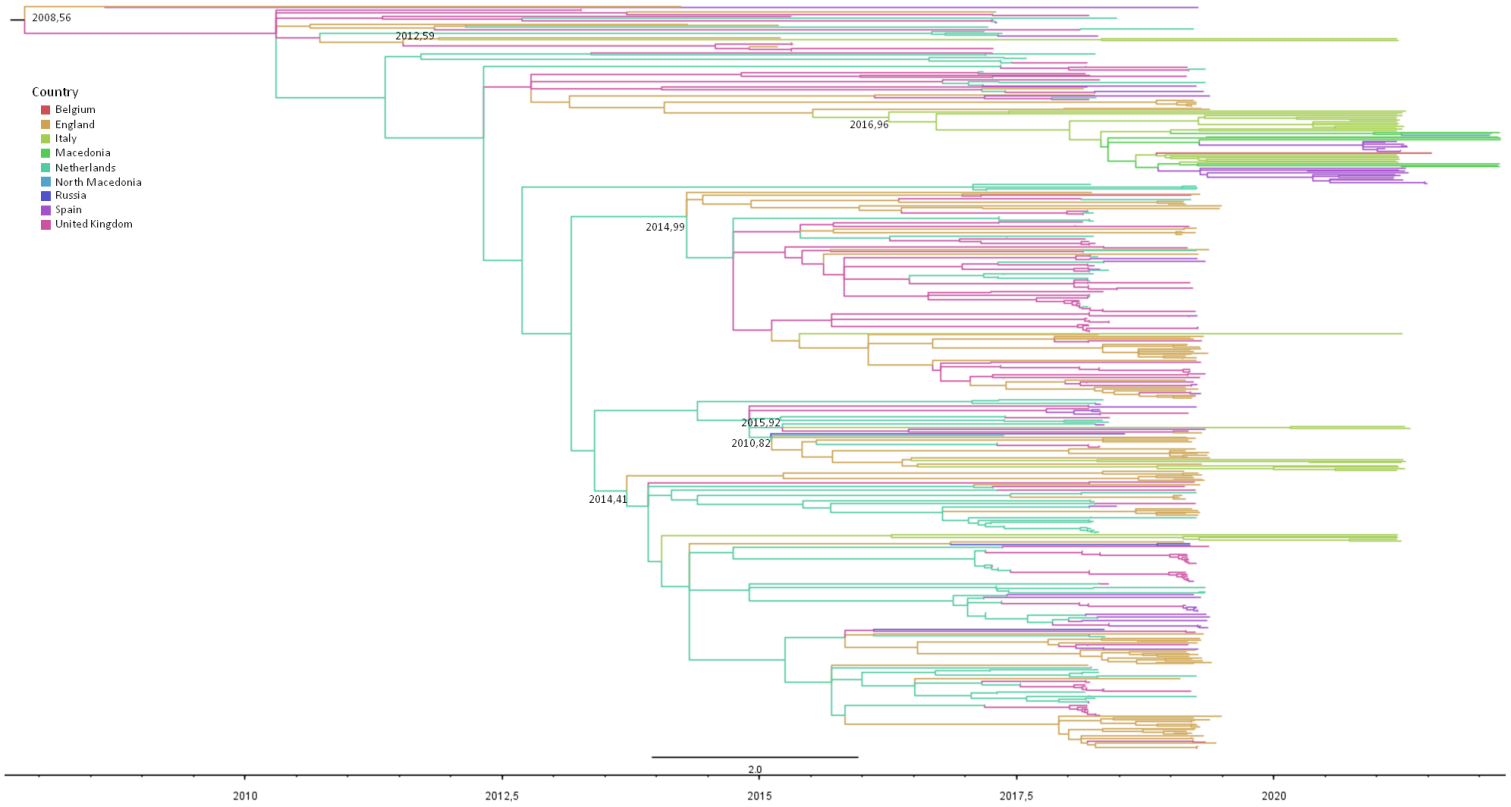
Maximum likelihood dated tree of RSV-B based on the whole genome. The branches’ colors represent the location (described in the legend). The time scale in fraction of year is detailed at the bottom. tMRCA of clusters involving Italians are reported.

Figure 3 shows the dated maximum likelihood tree of the RSV-B significant clusters. The tree root tMRCA corresponding to the hypothetical origin of GB5.0.5a lineage was estimated to be in average July 2008 (95% HPD: August 2007–February 2010). In particular, 21 out of 37 RSV-B Italian genomes (56.7%) were included in a single mixed cluster, including other European sequences (from the United Kingdom, Spain, the Netherlands, and Macedonia), whose tMRCA was estimated between May 2016 and June 2017 (mean estimate: December 2016). Nine isolates (37%) were included in two nested clusters (and #24) sharing a single MRCA dating between January 2015 and November 2015 (mean estimate: August 2015), encompassing also sequences obtained in the United Kingdom, the Netherlands, and Spain. Five Italian isolates (13.5%) were grouped into cluster #15, dating between January 2014 and January 2015 (average: May 2014). Two other patients’ sequences formed only a small clade of four sequences, while the last was a singleton (Table 4).

#### 3.6.3 Nextstrain analysis

The phylogeography of RSV-A and -B datasets was obtained by Nextstrain, a project allowing real-time snapshots of evolving pathogen populations, including ancestral state reconstruction and temporal inference by using TimeTree (the same program employed in our study).

The phylogenies did not show a significant geographic structure, probably because the genomes were sparsely sampled only in a few European countries.

#### 3.6.4 Population phylodynamic of RSV-A and RSV-B in Italy

The phylodynamic analysis was implemented on the main clusters, including the Italian sequences.

##### 3.6.4.1 RSV-A

**Figure 4:**
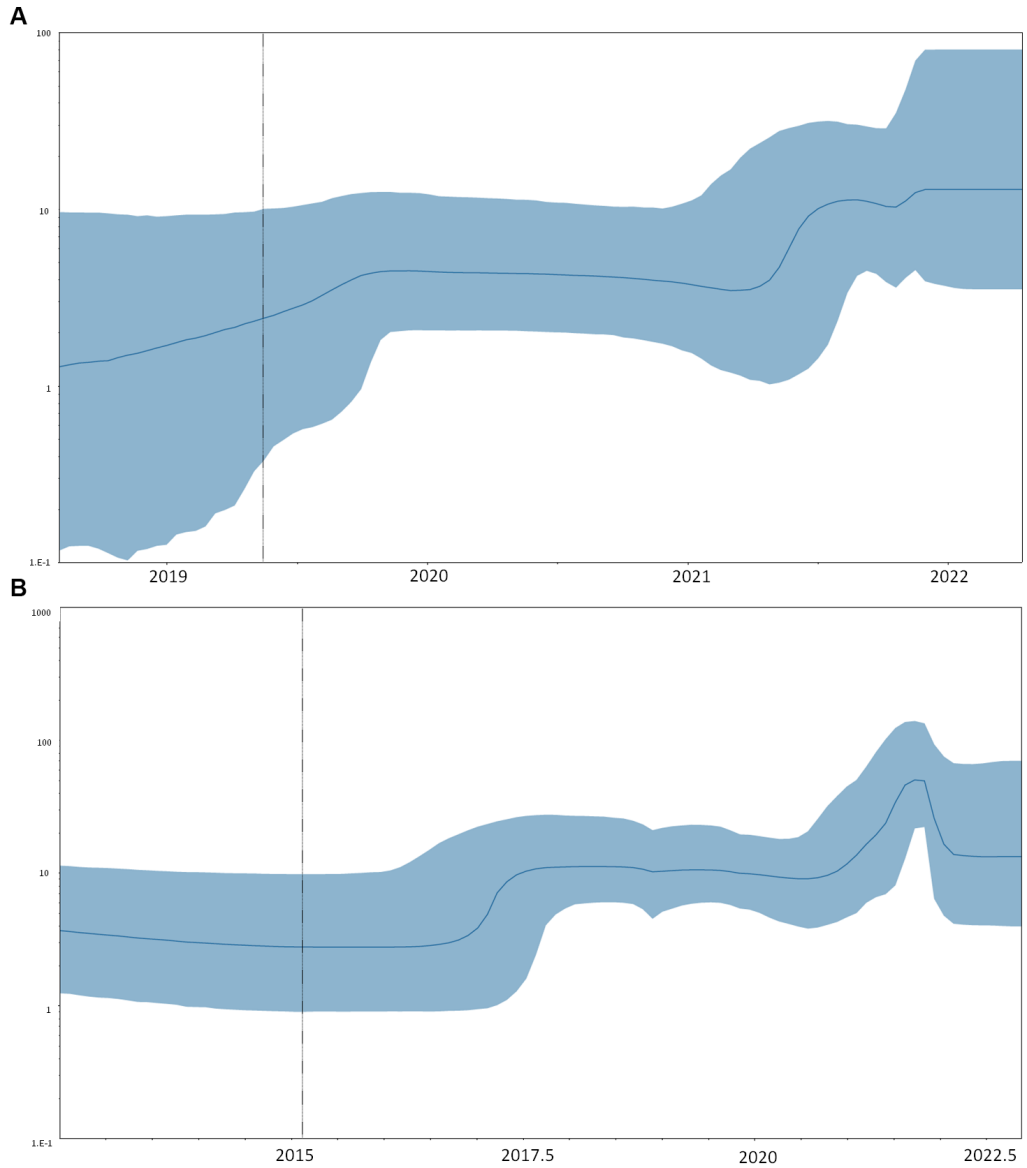
Bayesian skyline plot of RSV-A (A) and B (B). The *y*-axis indicates the effective population (Ne), the *x*-axis shows the time expressed in dates. The thick line in the graph indicates the median of the value of the estimate, while the blue area indicates 95% HPD.

The comparison between strict and relaxed clock models showed that the relaxed model was preferred (PS: −25865.0879 vs. −25849.1628, SS: −25865.3582 vs. −25844.4393 for strict and relaxed, respectively) with a mean evolutionary rate estimation of 0.94 (95% HPD: 0.64–1.22) × 10^−3^ sub/site/year for RSV-A. The skyline plot showed an initial growth of the curve representing the dynamic of clusters 25 and 26 between 2017 and 2018, corresponding to the root of the shared MRCA. A decline of the skyline plot was evidenced in the 2020–2021 season, followed by a rapid increase in late 2021 (Figure 4A).

##### 3.6.4.2 RSV-B

Even in this case, the relaxed clock was preferred to the strict one (PS: −34318.2806 vs. −34207.3695, SS: −34319.3784 vs. −34209.9933 for strict and relaxed, respectively), and a male evolutionary rate of 1.10 (95% HPD: 0.79–1.40) × 10^−3^ sub/site/year was estimated for RSV-B.

The skyline plot of RSV-B (Figure 4) showed the first growth of the transmission events between 2016 and 2017, corresponding to the tMRCA of the clusters, including Italian isolates. The curve shows a decrease in the number of infections during the year 2020, followed by a sharp spike in 2021 (Figure 4B).

## 4 Discussion

To study the genomic epidemiology of RSV in Italy, we enrolled a consecutive series of RSV-infected pediatric cases in a tertiary reference children’s hospital in Milan during the epidemic of the 2021–2022 season.

Currently, RSV is not an official notifiable infectious disease in Italy. Nevertheless, the ECDC recently recommended integrated respiratory virus surveillance (including influenza, SARS-CoV-2, RSV, and other important respiratory viruses) based on sentinel systems in primary and secondary care as a priority for the EU and WHO.^18^ In Italy, RSV surveillance has been implemented as part of the influenza-like illness (Wang et al., 2019, 2022) surveillance network (InfluNet), and some European countries reported data to the ECDC, allowing the evaluation of RSV activity in Europe in the last few years.

During the first year and a half of the pandemic, measures for mitigation and control of COVID-19 spread, such as social distancing, wearing masks, hygiene measures, travel restrictions, school closures, and lockdowns, led to a drastic reduction in respiratory viruses’ circulation, including RSV (Foley et al., 2021; Binns et al., 2022). Moreover, a decline in bronchiolitis cases and hospitalization has been observed in both the Northern and Southern hemispheres in the 2020–2021 season. Consequently, compared to the recent pre-COVID-19 period, the peak of the RSV epidemic was delayed by 2–9 months, determining an increase in the number of cases in the autumn of 2021 in several countries (Eden et al., 2022; Bardsley et al., 2023).

Despite most RSV infections result in relatively mild illness, age remains the most significant risk factor that increases the risk of severe RSV infection, as infants under 6 months are more likely to develop serious disease, particularly during their first 3 months of life. This is primarily because infants have smaller diameter airways, impaired respiratory capacity, and a lower respiratory reserve (Coultas et al., 2019), and the obstruction of the small airways has a greater clinical significance than an obstruction in the peripheral airways in an older child or in an adult (McNamara and Smyth, 2002). In the present study, we observed that hospitalization due to RSV infection was typically observed in children under 1 year of age, with the highest rate in children under 3 months of life (>80%).

Authors from different countries have reported an increase in the median age of infected children in the 2020–2021 season compared to previous seasons; in our study, the median age of patients is lower than that reported by Casalegno et al. (2021), McNab et al. (2021), and Yeoh et al. (2021).

Moreover, some studies hypothesized sex differences in respiratory viral pathogenesis and indicated a higher susceptibility to severe outcomes in male patients (Ursin and Klein, 2021). In our data, gender did not represent a risk factor for RSV infection, similar to the data reported by Tramuto et al. (2021).

Even though this is largely known, our study confirmed that infants who are hospitalized with a more severe disease due to RSV infection more often require respiratory support and a longer duration of oxygen support.

Surveillance studies on the molecular epidemiology of RSV have demonstrated that RSV subgroups may co-circulate during an epidemic with one that predominates over the other (Peret et al., 2000; Zlateva et al., 2007; Khor et al., 2013).

In Italy, data from the 2015–2016 to 2019–2020 seasons showed the prevalence of RSV-B, with the exception of 2019–2020 (Tramuto et al., 2021), while data from northern Italy indicated the predominance of RSV-B between the 2014–2015 and 2017–2018 seasons, followed by a 5-year RSV-A predominance (Esposito et al., 2015; Pellegrinelli et al., 2020). Our data indicated an equal proportion of infections sustained by each subgroup. This might also depend on the clinical features of the population included in the study. There is still debate on the possible different clinical presentation between RSV subtypes A and B, with some studies reporting that a different disease course may reflect different RSV genotypes and variants (Esposito et al., 2015; Rodriguez-Fernandez et al., 2017). In our study, after stratifying data by subgroups, RSV-A-positive cases were found to be older than the RSV-B-infected ones, and the overall clinical severity seemed to be higher. More molecular epidemiology studies are needed to understand whether the alternating predominance of RSV-A and RSV-B may be dictated by the introduction of novel genotypes/variants and if RSV antigenic diversity may have an impact on the hospitalization rate.

Several RSV genotypes and subgenotypes have been identified worldwide, with different regional and temporal distribution. In particular, different new genotypes appear periodically and tend to become dominant, replacing the oldest one (Goya et al., 2020; Ramaekers et al., 2020; Muñoz-Escalante et al., 2021). As genotype assignment, we adopted the criteria based on phylogenetic analysis and average p-distances of the G-ectodomain proposed by Goya et al. (2020). Our study showed that only two genotypes were present in all patients: the GA2 genotype of sub-genotype GA2.3.5, corresponding to the ON1 strain, among RSV-A-infected subjects and the genotype GB5 of sub-genotype GB5.0.5a, corresponding to a divergent BA strain, among RSV-B infected subjects. Both geno/subgenotypes are globally distributed and have become predominant in the last 15 years (Goya et al., 2020).

The maximum likelihood analysis of our sequences with a dataset of European genomes identified several significant clusters, including strains circulating all over the continent. Italian sequences were included in some mixed clusters and tended to concentrate on a few of them. The tMRCA estimation of these clusters showed that their origin went back to a period between 2011 and 2017. This suggests that the 2021–2022 outbreak in Italy was probably driven by multiple entry of clusters that had been circulating throughout Europe for at least 10 years and continued to circulate even during the period of pandemic restrictions, followed by the amplification of some of them due to local chains of infection.

The analysis of the genetic difference suggested a relatively limited nucleotide variability of RSV-A and -B genotypes, resulting in approximately 100 nucleotide substitutions in RSV-A and fewer (66) substitutions in RSV-B. The great majority of them were synonymous mutations, thus explaining the relatively low number of amino acid mutations observed in the viral proteins.

Clusters showed a mean intra-cluster distance lower than the mean overall estimation, showing a higher affinity between the strains included in the same cluster than between strains included in different ones. These mean differences were always lower than the limits suggested by Goya for the definition of new subgenotypes/clades (Goya et al., 2020; Ramaekers et al., 2020).

The *G* gene is the most variable gene, as confirmed by the distance between *G* sequences at least 2.5 times higher than that observed in the entire genomes in both subgroups; moreover, the non-synonymous mutations increased more than five times in the *G* gene than in whole genomes. This high variability was also confirmed by the great number of total amino acid substitutions (35 and 23 in subgroups A and B, respectively) and those under positive selection (3/3 in RSV-A and 5/6 in RSV-B) observed in G protein compared to more conserved F or L proteins. Nevertheless, the dN/dS ratio remained lower than 1 even in the *G* gene in both subgroups, suggesting a limited role of positive selection pressure even in this gene. This observation is partially in disagreement with the results obtained by Yu et al., who observed a dN/dS ratio > 1 in the *G* gene; this could be due to the presence in our study of strains of the same genotypes, thus suggesting that the positive selection pressure on the *G* gene is evident mainly at an inter-genotypic level (Yu et al., 2021).

None of the sites under significant positive selection previously described by other authors were found with the exception of codon 217 in RSV-B (Tan et al., 2012; Martinelli et al., 2014).

Some mutations, such as V279A in the *G* gene, N117K in the M2-1 portion, I7V and L422M in the *L* gene for HRSV-A and N5K in the NS2 portion, V90A in the *N* gene, T198A in the *G* gene, and A675V, UI677V, I1588, K1589, Y1590S, V1592L, and T1596I for HRSV-B, were present only in Italian strains.

Continued RSV evolution could also involve other antigenic sites of the F protein, which represents the main target protein for vaccine and anti-RSV antibody development. The prefusion F protein possessed highly neutralizing epitopes (site Ø, site III, site V, and site VIII), inducing potently neutralizing antibodies.

The substitutions found in our study were mainly located at sites Ø (206/209/211 position) and V (190/191 position) of RSV F in subgroup B, which were also observed in the previous studies (Bin et al., 2019; Sun et al., 2022). These mutations might affect viral antigenicity and facilitate immune escape of viruses. However, further studies are needed to determine the effects of these amino acid mutations at RSV-neutralizing epitopes.

Moreover, as passive immunization with palivizumab reduces the risk in vulnerable infants of acquiring RSV, the finding of palivizumab-resistant viruses might be of clinical relevance. In the region encompassing amino acids 262–276 of the F protein, that is, the drug-binding region, we did not observe mutations in both subgroups. Similarly, we did not find substitutions related to resistance at suptavumab (Simões et al., 2021) and/or nirsevimab (Peeples and Thongpan, 2023).

The analysis of the population dynamics was performed on RSV-A and -B clusters, including the majority of the Italian strains. In both cases, the skyline plot showed a first growth in the period between 2016 and 2017, corresponding to the first expansion of the clusters, followed by a period of constant size or a slight decrease in 2020–2021 and then a sharp increase in 2021–2022. This suggests that the strains that gave rise to the 21–22 outbreak in Italy continued to circulate while experiencing a partial contraction in the number of infections during the period when pandemic control measures were in place and then exploded when control measures were discontinued.

The spatial and temporal distribution of RSV is currently analyzed using novel real-time surveillance tools, such as Nextstrain (Hadfield et al., 2018).

It is important to note that we did not observe a strong spatial structure in the tree, with Ga2.3.5 and GB5.0.5a co-circulating globally. In light of these findings, it appears that the evolution of RSV-A and -B was not strongly regionalized.

The main limitation of our study is that it is based on a retrospective analysis of a single children’s hospital, thus limiting the value of our speculations.

## 5 Conclusion

In 2021, the explosive increase in bronchiolitis cases requiring hospitalization was caused by viral strains of RSV-A and RSV-B (at similar frequencies), belonging to the most prevalent subgenotypes, included in clusters circulating in Europe since at least 2016–2017. It is difficult to estimate how long these clusters have existed in Italy, given the lack of Italian sequences collected before 2021 and the limited phylogeographic information available on the sequences included. Therefore, we cannot speculate on whether the epidemic was the result of multiple introductions from outside Italy or of the spread of clusters already present in the country. It can therefore be hypothesized that the high number of RSV cases observed in the fall of 2021 may be related, on the one hand, to the relaxation of COVID-19 pandemic control measures and, on the other hand, to the increased risk of infection in a high-risk population more exposed to clinical sequelae of RSV infection, such as infants who have been previously indirectly protected by the reduction of infections from parents and siblings. While further studies on this topic would be desirable, this study adds new data on the molecular epidemiology of RSV in Italy, highlighting its importance in support of the existing respiratory pathogens’ surveillance systems both in the community and in hospital settings. Global molecular epidemiology of RSV is important for detecting the emergence and spread of new strains, predicting their clinical impact, and understanding their dynamic evolution in future. Since the evolution of RSV strains is a continuous process, with relatively rapid sequential replacement of dominating strains approximately every 7 years (Otieno et al., 2016), the availability of more whole virus genome sequences from recent isolates representing the diversity of the circulating RSV population will provide valuable information to improve our knowledge on the epidemiology and evolutionary dynamics of the viruses, as well as to develop effective vaccines and/or therapeutics.

## Data availability statement

All consensus genomes are submitted at the GISAID database (EPI_ISL_18446761-EPI_ISL_18446848). All the files used for the analyses are available upon request.

## Ethics statement

For all participants, informed consent was obtained from the parents or legal guardians. All data used in this study were previously anonymized as required by the Italian Data Protection Code (Legislative Decree 196/2003) and the general authorizations issued by the Data Protection Authority. The study was conducted in compliance with Good Clinical Practice (https://ichgcp.net/it) and the Declaration of Helsinki.

## Author contributions

AL: Conceptualization, Data curation, Formal Analysis, Investigation, Methodology, Supervision, Visualization, Writing – original draft, Writing – review & editing. AB: Conceptualization, Data curation, Investigation, Methodology, Visualization, Writing – original draft, Writing – review & editing. VF: Conceptualization, Data curation, Visualization, Writing – review & editing. CdV: Data curation, Investigation, Visualization, Writing – review & editing. GF: Data curation, Investigation, Visualization, Writing – review & editing. AM: Data curation, Visualization, Writing – review & editing. ML: Data curation, Visualization, Writing – review & editing. GVZ: Conceptualization, Visualization, Writing – review & editing. GZ: Conceptualization, Data curation, Funding acquisition, Investigation, Visualization, Writing – original draft, Writing – review & editing.

## Footnotes

^1^ https://www.niaid.nih.gov/diseases-conditions/respiratory-syncytial-virus-rsv

^2^ https://www.ecdc.europa.eu/en/publications-data/intensified-circulation-respiratory-syncytial-virus-rsv-and-associated-hospital

^3^ https://www.epicentro.iss.it/influenza/influnet

^4^https://ichgcp.net/it.

^5^http://www.geneious.com, Biomatters, Auckland, New Zealand.

^6^ https://clades.nextstrain.org/

^7^ https://gisaid.org

^8^ https://www.ncbi.nlm.nih.gov/genbank/

^9^ https://mafft.cbrc.jp/alignment/server/

^10^ http://www.mbio.ncsu.edu/bioedit/bioedit.html

^11^ http://www.iqtree.org/

^12^ http://tree.bio.ed.ac.uk/software/figtree/

^13^ http://tree.bio.ed.ac.uk/software/tempest/

^14^ https://www.datamonkey.org/

^15^ https://beast.community/

^16^ http://tree.bio.ed.ac.uk/software/tracer/

^18^ https://www.who.int/europe/publications/i/item/WHO-EURO-2022-5841-45606-65427
