# Supplementary material for: Epidemiology and molecular analyses of respiratory syncytial virus in the 2021–2022 season in northern Italy
Source: Front Microbiol. 2024 Jan 4;14:1327239. doi: 10.3389/fmicb.2023.1327239 (PMC10794773; doi:10.3389/fmicb.2023.1327239)
Supplement: Supplementary file 1 [file Table_1.DOCX]

Supplementary Material

# Supplementary Table

**Table S1**. Amino acid substitutions found in RSV-A and B dataset.

**A**

| **Gene** | **Substitution** | **Total dataset n=359** | **Italians n=49** | **Europeans n=310** | **# Cluster** |
| --- | --- | --- | --- | --- | --- |
|  |  | **%(n)** | **%(n)** | **%(n)** |  |
| *NS1* (139 aa) | V82I | 1.9 (7) | 10.2 (5) | 0.6 (2) |  |
| *N* (391 aa) | V352A | 98.6 (354) | 100 (49) | 98.4 (305) | 5, 12, 14, 25, 26 |
| *P* (241 aa) | T69I | 8.6 (31) | 53.1 (26) | 1.6 (5) |  |
|  | T92M | 15.0 (54) | 16.3 (8) | 14.8 (46) |  |
| *M* (256 aa) | M43I | 35.4 (127) | 2.0 (1) | 40.6 (126) |  |
|  | M73L | 14.2 (51) | 77.6 (38) | 4.2 (13) | 25, 26 |
| *SH* (64 aa) | I38S | 15.3 (55) | 0 (0) | 17.7 (55) |  |
| *G* (298 aa) | A57V | 14.2 (51) | 77.6 (38) | 4.2 (13) | 25, 26 |
|  | P71L | 96.7 (347) | 100 (49) | 96.1 (298) | 12, 14, 25, 26 |
|  | H90Y | 96.9 (348) | 98.0 (48) | 96.8 (300) | 12, 14, 25, 26 |
|  | S100I | 11.4 (41) | 0 (0) | 13.2 (41) |  |
|  | L101F | 93.9 (337) | 98.0 (48) | 93.2 (289) | 12, 14, 25, 26 |
|  | G106E | 9.7 (35) | 0 (0) | 11.3 (35) |  |
|  | T113I | 15.3 (55) | 16.3 (8) | 15.2 (47) |  |
|  | P120L | 1.7 (6) | 10.2 (5) | 0.3 (1) |  |
|  | V131D | 15.6 (56) | 16.3 (8) | 15.5 (48) |  |
|  | I134K | 85.2 (306) | 98.0 (48) | 83.2 (258) | 12, 14, 25, 26 |
|  | L142S | 35.7 (128) | 6.1 (3) | 40.3 (125) |  |
|  | N178G | 15.0 (54) | 16.3 (8) | 14.8 (46) |  |
|  | P206Q | 19.8 (71) | 79.6 (39) | 10.3 (32) | 12, 25, 26 |
|  | T207N | 2.2 (8) | 12.2 (6) | 0.6 (2) |  |
|  | K209R | 15.3 (55) | 77.6 (38) | 5.5 (17) | 25, 26 |
|  | K212E | 10.6 (38) | 0 (0) | 12.3 (38) |  |
|  | G224E | 91.1 (327) | 98.0 (48) | 90.0 (279) | 12, 14, 25, 26 |
|  | S243I | 89.7 (322) | 98.0 (48) | 88.4 (274) | 12, 14, 26 |
|  | L248I | 17.5 (63) | 79.6 (39) | 7.7 (24) | 12, 25, 26 |
|  | H258Q | 15.6 (56) | 16.3 (8) | 15.5 (48) |  |
|  | K262E | 81.9 (294) | 95.9 (47) | 79.7 (247) | 12, 14 |
|  | I265L | 88.6 (318) | 98.0 (48) | 87.1 (270) | 12, 14 |
|  | H266L | 14.8 (53) | 16.3 (8) | 14.5 (45) |  |
|  | S270P | 13.1 (47) | 0 (0) | 15.2 (47) |  |
|  | Y273H | 5.8 (21) | 18.4 (9) | 3.9 (12) |  |
|  | P274L | 29.8 (107) | 16.3 (8) | 31.9 (99) | 14 |
|  | V279A | 10.9 (39) | 79.6 (39) | 0 (0) |  |
|  | Y280H | 18.1 (65) | 79.6 (39) | 8.4 (26) |  |
|  | D284G | 77.7 (279) | 0 (0) | 90.0 (279) |  |
|  | P298L | 20.6 (74) | 0 (0) | 23.9 (74) |  |
|  | Y304H | 46.8 (168) | 0 (0) | 54.2 (168) |  |
|  | L310P | 26.5 (95) | 79.6 (39) | 18.1 (56) | 12, 25 |
|  | S316L | 10.6 (38) | 0 (0) | 12.3 (38) |  |
|  | T319I | 17.3 (62) | 77.6 (38) | 7.7 (24) | 25 |
|  | T320A | 48.7 (175) | 81.6 (40) | 43.5 (135) | 12, 25 |
| *F* (574 aa) | T12I | 15.3 (55) | 16.3 (8) | 15.2 (47) |  |
|  | A103T | 8.1 (29) | 53.1 (26) | 1.0 (3) |  |
|  | M115T | 2.2 (8) | 12.2 (6) | 0.6 (2) |  |
|  | T122A | 19.8 (71) | 79.6 (39) | 10.3 (32) | 12, 25, 26 |
| *M2-1* (194 aa) | N117K | 1.4 (5) | 10.2 (5) | 0 (0) |  |
|  | S176P | 96.7 (347) | 98.0 (48) | 96.5 (299) | 14 |
| *M2-2* (88 aa) | S46N | 79.4 (285) | 98.0 (48) | 76.5 (237) | 5, 14, 26 |
|  | T79A | 14.8 (53) | 16.3 (8) | 14.5 (45) |  |
| *L* (2165 aa) | I7V | 1.7 (6) | 12.2 (6) | 0 (0) |  |
|  | N146D | 31.2 (112) | 0 (0) | 36.1 (112) |  |
|  | P174L | 93.6 (336) | 98.0 (48) | 92.9 (288) | 14, 25, 26 |
|  | T182S | 30.1 (108) | 0 (0) | 34.8 (108) |  |
|  | R259K | 93.3 (335) | 98.0 (48) | 92.6 (287) | 12, 14, 25 |
|  | L422M | 1.4 (5) | 10.2 (5) | 0 (0) |  |
|  | Y601H | 85.0 (305) | 98.0 (48) | 82.9 | 12 |
|  | L838M | 15.0 (54) | 18.4 (9) | 14.5 (45) |  |
|  | K1028R | 2.2 (8) | 14.3 (7) | 0.3 (1) |  |
|  | L1441Q | 96.9 (348) | 98.0 (48) | 96.8 (300) | 12, 14, 26 |
|  | I1656V | 31.2 (112) | 0 (0) | 36.1 (112) |  |
|  | K1664N | 30.9 (111) | 0 (0) | 35.8 (111) |  |
|  | N1726G | 17.0 (61) | 75.5 (37) | 7.7 (24) | 12, 14 |
|  | N1726S | 75.8 (272) | 20.4 (10) | 84.5 (262) |  |
|  | E1728G | 63.0 (226) | 63.3 (31) | 62.9 (195) |  |
|  | G1734D | 88.6 (318) | 95.9 (47) | 87.4 (271) | 12, 14, 26 |
|  | V1946D | 17.0 (61) | 77.6 (38) | 7.4 (23) | 12, 26 |
|  | N2114H | 11.4 (41) | 0 (0) | 13.2 (41) |  |
|  | Y2166N | 15.0 (54) | 0 (0) | 17.4 (54) |  |

**B**

| **Gene** | **Substitution** | **Total dataset n=806 % (n)** | **Italians n=39 % (n)** | **Europeans n=767 % (n)** | **# Cluster** |
| --- | --- | --- | --- | --- | --- |
| *NS2* (124 aa) | N5K | 1.5 (12) | 30.8 (12) | 0 (0) |  |
| *N* (391 aa) | V90A | 0.8 (7) | 18 (7) | 0 (0) |  |
|  | V97I | 95.8 (772) | 100 (39) | 95.6 (733) | 2, 15, 23, 24, 28 |
| *M* (256 aa) | T89I | 1.5 (12) | 12.9 (5) | 0.9 (7) |  |
| *SH* (64 aa) | D64N | 16.6 (134) | 5.1 (2) | 17.2 (132) |  |
| *G* (298 aa) | A74V | 99.9 (805) | 97.4 (38) | 100 (767) | 13, 15, 23, 24, 28 |
|  | S100G | 8.1 (65) | 51.3 (20) | 5.9 (45) |  |
|  | Q104H | 0.6 (5) | 10.3 (4) | 0.1 (1) |  |
|  | H128Y | 0.6 (5) | 10.3 (4) | 0.1 (1) |  |
|  | T131A | 46.8 (377) | 56.4 (22) | 46.3 (355) |  |
|  | G135S | 4.1 (33) | 10.3 (4) | 3.8 (29) |  |
|  | I137T | 46.2 (372) | 56.4 (22) | 45.6 (350) |  |
|  | T141K | 0.6 (5) | 10.3 (4) | 0.1 (1) |  |
|  | T198A | 0.5 (4) | 10.3 (4) | 0 (0) |  |
|  | P214S | 6.5 (52) | 51.3 (20) | 4.2 (32) |  |
|  | L217P | 16 (129) | 33.3 (13) | 15.1 (116) |  |
|  | P221L | 6.5 (52) | 51.3 (20) | 4.2 (32) |  |
|  | P229L | 15.1 (122) | 0 (0) | 16 (122) |  |
|  | P235L | 1.7 (14) | 12.8 (5) | 1.2 (9) |  |
|  | D251N | 3 (24) | 12.8 (5) | 2.5 (19) |  |
|  | I252T | 14 (113) | 59 (23) | 11.7 (90) |  |
|  | K256N | 3.7 (30) | 48.7 (19) | 1.4 (11) |  |
|  | S265P | 6.6 (53) | 12.8 (5) | 6.3 (48) |  |
|  | I268T | 11.2 (90) | 56.4 (22) | 8.9 (68) |  |
|  | S275P | 3.6 (29) | 48.7 (19) | 1.3 (10) |  |
|  | Y285H | 18.7 (151) | 51.3 (20) | 17.1 (131) |  |
|  | I288T | 15.6 (126) | 5.1 (2) | 16.2 (124) |  |
|  | I310T | 35.6 (287) | 5.1 (2) | 37.2 (285) |  |
| *F* (574 aa) | A19V | 0.6 (5) | 10.3 (4) | 0.1 (1) |  |
|  | S190N | 7.6 (61) | 53.8 (21) | 5.2 (40) | 28 |
|  | R191K | 15.5 (125) | 7.7 (3) | 16 (122) | 2 |
|  | M206I | 15.5 (125) | 7.7 (3) | 16 (122) | 2 |
|  | R209Q | 16.1 (130) | 12.8 (5) | 16.3 (125) | 2 |
|  | S211N | 6.9 (56) | 53.8 (21) | 4.6 (35) | 28 |
|  | S389P | 6.3 (51) | 53.8 (21) | 3.9 (30) | 28 |
|  | M526V | 0.6 (5) | 10.2 (4) | 0.1 (1) |  |
| *M2-2* (88 aa) | I2T | 97.9 (789) | 97.4 (38) | 98 (751) | 2, 23, 28 |
|  | M27T | 25.9 (208) | 53.8 (21) | 24.4 (187) |  |
|  | D35N | 6.5 (52) | 53.8 (21) | 4 (31) | 28 |
|  | C49F | 8.3 (67) | 53.8 (21) | 6 (46) | 28 |
| *L* (2165 aa) | K570R | 8.3 (67) | 53.8 (21) | 6 (46) | 28 |
|  | A675V | 0.8 (7) | 18 (7) | 0 (0) |  |
|  | I677V | 0.6 (5) | 12.8 (5) | 0 (0) |  |
|  | A1479V | 14 (113) | 5.1 (2) | 14.5 (111) |  |
|  | I1588L | 0.5 (4) | 10.3 (4) | 0 (0) |  |
|  | K1589M | 0.5 (4) | 10.3 (4) | 0 (0) |  |
|  | Y1590S | 0.5 (4) | 10.3 (4) | 0 (0) |  |
|  | V1592L | 0.5 (4) | 10.3 (4) | 0 (0) |  |
|  | T1596I | 0.7 (6) | 15.4 (6) | 0 (0) |  |
|  | V1716I | 12.5 (101) | 10.2 (4) | 12.6 (97) | 2 |
|  | R1759K | 6.2 (50) | 48.7 (19) | 4 (31) |  |
|  | V1965A | 0.6 (5) | 10.3 (4) | 0.1 (1) |  |
|  | T1987I | 80.8 (651) | 66.7 (26) | 81.5 (625) | 2 |
